# Chemoselective Coatings of GL13K Antimicrobial Peptides for Dental Implants

**DOI:** 10.3390/pharmaceutics15102418

**Published:** 2023-10-04

**Authors:** Isha Mutreja, Caixia Lan, Qishun Li, Conrado Aparicio

**Affiliations:** 1 MDRCBB−Minnesota Dental Research Center for Biomaterials and Biomechanics, Minneapolis, MN 55455, USA; imutreja@umn.edu (I.M.); lqsdoctor@163.com (Q.L.); 2 The Affiliated Stomatological Hospital of Nanchang University, Nanchang 330000, China; 3 Faculty of Odontology, UIC Barcelona−International University of Catalonia, 08198 Sant Cugat del Vallès, Spain; 4 IBEC Institute for Bioengineering of Catalonia, 08170 Barcelona, Spain

**Keywords:** antimicrobial peptide, surface coating, dental implants, biocompatibility, titanium

## Abstract

Dental implant−associated infection is a clinical challenge which poses a significant healthcare and socio−economic burden. To overcome this issue, developing antimicrobial surfaces, including antimicrobial peptide coatings, has gained great attention. Different physical and chemical routes have been used to obtain these biofunctional coatings, which in turn might have a direct influence on their bioactivity and functionality. In this study, we present a silane−based, fast, and efficient chemoselective conjugation of antimicrobial peptides (Cys−GL13K) to coat titanium implant surfaces. Comprehensive surface analysis was performed to confirm the surface functionalization of as−prepared and mechanically challenged coatings. The antibacterial potency of the evaluated surfaces was confirmed against both Streptococcus gordonii and Streptococcus mutans, the primary colonizers and pathogens of dental surfaces, as demonstrated by reduced bacteria viability. Additionally, human dental pulp stem cells demonstrated long−term viability when cultured on Cys−GL13K−grafted titanium surfaces. Cell functionality and antimicrobial capability against multi−species need to be studied further; however, our results confirmed that the proposed chemistry for chemoselective peptide anchoring is a valid alternative to traditional site−unspecific anchoring methods and offers opportunities to modify varying biomaterial surfaces to form potent bioactive coatings with multiple functionalities to prevent infection.

## 1. Introduction

The utilization of dental implants to replace missing teeth has become the preferred alternative for functional and aesthetic purposes [1,2,3,4,5]. The dental implant market was valued at USD 4.6 billion in 2022, which is predicted to grow at a compound annual rate of 9.8% from 2023 to 2030. Despite their increased utilization and high 10−year survival rates, there have been increased instances of patients suffering from peri−implant diseases affecting the integrity of both soft and hard tissue. A recent long−term follow−up study suggested that on an average of 23.3 years of treatment with dental implants, approximately 54.7% of patients suffered from peri−implant mucositis, and nearly 22.15% had peri−implantitis [6]. Clinically, peri−implantitis is characterized by inflammation in the peri−implant mucosa and the progressive loss of the supporting bone, which if left untreated results in implant failure [6,7,8].

Titanium−based materials have been the implant materials of choice for decades due to their mechanical properties, biocompatibility, and corrosion resistance [2,9,10,11]. Additionally, these properties make these materials suitable for bacterial colonization and growth. Implant−associated infection can arise at any time, but the first four weeks post implantation are the most vulnerable, as the interface between the implant and the surrounding bone is weak [10]. Therefore, efforts have been focused on designing strategies to impart anti−infective properties to the implant surface to arrest bacterial colonization on the surface [11,12,13,14]. Most of these modifications rely on restricting bacterial adhesion (anti−adhesive), reducing colonization (antibacterial and thus anti−proliferative), or targeting biofilm formation. These include the bioinspired texturing of the implant surface to form a ‘bed of nails’ [15], like fabricating nanopillars or nanospikes, or mimicking structures present on the wings of a dragonfly [16]. Apart from the surface structure−mediated anti−infective strategies, alternative methods of delivering bactericidal agents at the surface of the implant as a dynamic approach are being investigated as well [4]. Some of these approaches include modifying the surface with a thin coating which releases antibiotics like vancomycin [17] or chlorhexidine [18], antibacterial polymers [3,19,20], metal ions or metallic nanoparticles [21,22,23], biofilm inhibitors relying on inducers like Methylthioadenosine nucleosidase (MTAN) involved in quorum sensing [24,25] and antimicrobial peptides—AMPs [1,21,26,27,28]. Alternatively, the surface of the implant is electrochemically anodized to serve as a reservoir for the functional agents, like antibiotics [4,29,30], or metallic or semi−metallic ions or nanoparticles like silver, gold, copper, zinc, or gallium [31] to impart strong bactericidal function.

Despite the growing interest in modifying the surface of the implant to passively control bacterial colonization via a ‘bed of nails’, or by forming nanotubes/nanopores for improved osseointegration and minimizing infection, the long−term mechanical stability of these features to sustain manufacturing and clinical handleability during implantation and during its contact with the bone needs further investigation [32,33]. Therefore, coating strategies which rely on chemically immobilizing the bioactive agent onto the surface without introducing significant changes in the surface structure offer a lot of potential. Our previous work has demonstrated the long−term stability and functional efficacy of the bioactive coatings, even after mechanical challenges [1,34]. Additionally, the coating strategy can be easily translated onto different materials. This is particularly important as there is a gradual shift from traditional cpTi−based dental implants to using other materials as well, like titanium alloy [35,36,37,38], PEEK [39,40,41], or zirconia [42,43]. Corrosion resistance, excellent biocompatibility, and mechanical properties are the key properties that make titanium and its alloys the materials of choice for dental implants. However, the mechanical mismatch between the metal/metallic alloy and the surrounding bone is one of the most common causes of implant failure due to insufficient stress shielding and bone resorption. On the other hand, Zirconia is a ceramic material that is gaining popularity due to its biocompatibility, corrosion resistance, and aesthetic properties. On the contrary, PEEK is a synthetic material that offers the same advantageous properties as titanium and titanium alloy but matches the tooth aesthetic properties along with the mechanical properties of the surrounding cortical bone.

For designing these antibacterial coatings, different agents like metallic nanoparticles, antibiotics, or AMPs can be used. Amongst these agents, AMPs have gained significant interest. AMPs generally demonstrate broad−spectrum microbicidal activity with efficacy against potent bacterial strains like methicillin−resistant *Staphylococcus aureus* [44,45]. The claimed mode of action for AMPs is non−specific membrane targeting [46]; thus, they offer a viable option for overcoming antibiotic resistance, including when immobilized on implant, device, and particle surfaces [46].

One such designer of AMPs, GL13K derived from the human salivary parotid secretory protein (BPIFA2), has demonstrated broad−range antibacterial efficacy against Gram−negative and Gram−positive bacteria, low bacterial resistance, and low cytotoxicity [1,34,47,48,49,50,51,52,53,54,55]. This peptide has shown efficacy both in solution [54] and after surface immobilization [1,21,34,49]. Different techniques have been employed for coating a wider range of surfaces, which have either relied on the self−assembly of the peptide under alkaline conditions [21,56], or covalent immobilization using silanization [1,34,49]. These methods have demonstrated the durability of the coated surfaces [1,49], and even potency after incubation with highly cariogenic biofilms [57]. However, the previously mentioned silanization method relies on the free amines in the lysine residues present in the GL13K peptide. Consuming the lysine residues reduces the overall positive charges, which may impact the bioactivity of the immobilized peptides. Previous reports relying on this method for immobilizing GL13K on titanium did not demonstrate any impact on the antibacterial efficacy of the peptides, but it is unclear if this response will be altered based on the nature of the substrate. In addition, due to the competitive nature of the reaction and no control as to which lysine will be involved in substrate anchoring, the conjugation process is less controllable.

Herein, we propose an alternative mode of surface immobilization for AMP GL13K peptides which relies on a thiol–maleimide−based Michael−type addition reaction [58,59,60,61,62]. The high specificity between maleimide and free thiol provides high chemical selectivity and conjugation efficiency. In addition, the fast reaction kinetics and the feasibility of performing the reaction at near−neutral pH conditions in the absence of the catalyst at room temperature make it an attractive strategy to modify a range of substrates like metals, polymers, and nanoparticles.

To do so, GL13K peptide with an added cysteine residue at the N−terminus was used for the chemoselective reaction with a maleimide−functionalized titanium surface. Here, we demonstrated the ability of this conjugation chemistry to form a potent antimicrobial coating. Furthermore, the durability, functionality, and cytocompatibility of the chemoselective antimicrobial peptide coating were investigated.

## 2. Materials and Methods

***Materials:*** Cysteine−substituted GL13K peptide Cys−GL13K (CGKIIKLKASLKLL−CONH_2_; MW: 1545 gmol^−1^) and unmodified L−GL13K (GKIIKLKASLKLL−CONH_2_; MW: 1424 gmol^−1^) were purchased from Apeptide (Shanghai, China) with a purity of >98%. Ti disks (7 mm diameter) were punched from sheets of commercially pure Grade II Ti (cp Ti; McMaster−Carr; Robbinsville, NJ, USA). (3−Aminopropyl)triethoxysilane (APTES) was purchased from Sigma−Aldrich ((Burlington, MA, USA). Sulfosuccinimidyl−4−(N−maleimidomethyl)cyclohexane−1−carboxylate (Sulfo−SMCC), minimum essential medium α (α−MEM), penicillin–streptomycin (Pen−Strep), fetal bovine serum (FBS), and phosphate buffer saline (PBS) were purchased from ThermoFisher Scientific (Waltham, MA, USA). Brain heart infusion (BHI), Todd–Hewitt broth (THB) powders, and other bacterial culture relative agars were from BD Biosciences (San Jose, CA, USA). The bacterial strain Streptococcus gordonii (*S. gordonii*; ML−5) and Streptococcus mutans (S. mutans; ATCC 700610) were used. Human dental pulp stem cells were kindly donated by Kim Mansky’s lab at the University of Minnesota.

**Structural confirmation of Cys−GL13K and L−GL13K:** Circular dichroism (CD) analysis was performed to analyze the peptide conformation at neutral and basic pH conditions. To conduct this, peptide solutions were prepared in PBS (pH 7.4) at the concentration of 0.5 mg/mL. A JASCO J−815 CD spectropolarimeter was used to record the spectra of the peptides using a quartz cell with 0.1 cm path length. CD analysis was carried out over the wavelength ranging from 190 nm to 260 nm. The CD spectra of the two peptides were recorded at pH 7.4, after which the pH was adjusted to 9.5 to analyze the change in peptide conformation with pH. CD data were presented as the mean residue ellipticity and the data were analyzed using a set of software programs (CDSSTR, CONTINLL and SELCON3) in CD Pro Analysis to determine the percentage of the different secondary structure components.

**Figure 1:**
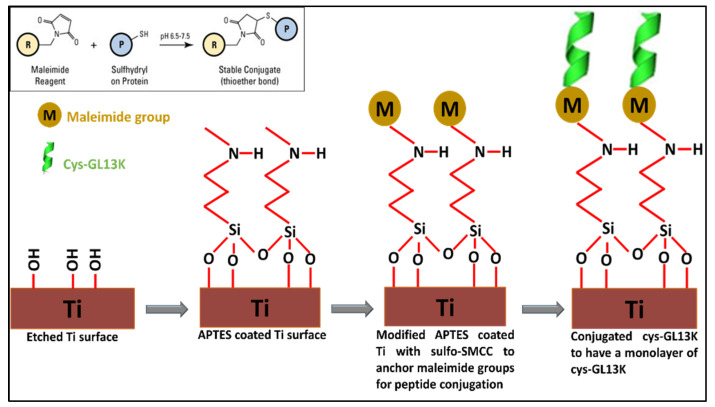
Schematic of peptide coating on eTi surface via thiol–maleimide coupling.

**Peptide coating on eTi surface:** The titanium (eTi) surfaces were coated with cys−GL13K by modifying a previously published protocol (43). The schematic is represented in Figure 1. Briefly, polished Ti discs (ø 7 mm) were etched in 5 M NaOH solution at 60 °C overnight to activate the surface with –OH groups. Post etching, the samples were thoroughly cleaned and silanized with 5% APTES solution (dissolved in 95% ethanol) at 60 °C for 2 h. After silanization, the samples were cleaned, and the surface was modified with maleimide. This was achieved by immersing the silanized samples in 0.5 mg/mL sulfo−SMCC solution (prepared in PBS, pH 7.4) for 30 min at room temperature. Post maleimide modification, the samples were coated with cys−GL13K by immersion in 0.5 mM peptide solution (pH 7.4) for 30 min. The samples were washed thoroughly using distilled water, acetone, and ethanol.

**Characterization of the peptide coating:** The surface chemistry and composition of the samples were characterized by Fourier−transform infrared (FT−IR) spectroscopy, X−ray photoelectron spectroscopy (XPS), and water contact angle measurements. ATR (attenuated total reflectance) mode was used on the FT−IR spectrometer (Nicolet iS50, Thermo Fisher Scientific) to record the spectra of the samples in the range of 400 to 4000 cm^−1^ with an incremental step size of 2 cm^−1^. The spectral average was collected over 64 scans per sample. For XPS, a PHI 5000 VersaProbe III (ULVAC Inc, Kanagawa, Japan) was used to measure surface elemental composition. The system used a monochromatic Al Kα X−ray source (45°, 1486.6 eV, 50 W, sampling area; 200 μm diameter). A spectral survey was collected at a pass energy of 280 eV with a step size of 1.0 eV. MultiPak (PHI, ULVAC Inc, Kanagawa, Japan) was used to determine the %atomic composition after background subtraction. The change in surface wettability was determined using the sessile drop (deionized water; 2 μL) method using a contact angle goniometer (DM−CE1, Kyowa Interface Science, Japan) and FAMAS software (Kyowa Interface Science, Japan).

**Durability of the peptide coating:** Fluorescently tagged cys−GL13K−FAM peptide was used for this experiment. The labelled peptide was immobilized either covalently (experimental group) using the method mentioned in the previous section, or physiosorbed (control) on silanized Ti surfaces without a maleimide coating. The durability of the control and the experimental groups was determined by mechanically challenging the samples by ultrasonication in deionized water for short (5 min) or long (30 min) durations. The as−prepared and challenged samples were washed and micrographs were acquired on an upright fluorescent microscope (Eclipse E800, Nikon, Japan). Additionally, the change in the contact angle of the mechanically challenged coatings was also determined.

**Antibiofilm activity against *S. gordonii* and *S. mutans*:** The groups tested in this study included discs that were etched, silanized, maleimide−coated, and cys−GL13K−coated. Samples were disinfected with 70% ethanol by immersing them in the disinfectant for 30 min, after which they were rinsed in autoclaved deionized water, blot−dried on sterile gauze, and lastly, UV−treated for 40 min prior to bacterial culture. Overnight, the inoculum of *S.mutans* was diluted to an OD value of 0.6 and after adjusting the OD, the culture was further diluted 25−fold using BHI media. For *S. gordonii,* the overnight inoculum was diluted 1:2000 in THB media. For both the bacterial strains, 1 mL of diluted inoculum was added to each sample and samples were incubated for 20 h in an anaerobic chamber at 37 °C with gentle shaking.

Bacterial viability and metabolic activity were determined by counting colony forming units (CFUs) and measuring adenosine triphosphate (ATP) activity. For this, after 20 h culture, samples were washed gently using 0.9% sodium chloride (NaCl) solution three times. After washing, the biofilm was detached from the sample in 1 mL of 0.9% NaCl solution by sonication using an ultrasonic scaler (Cavitron Select SPS ultrasonic scaler type Gen−124, Dentsply Sirona, Charlotte, NC, USA) without water irrigation. To perform this, the sample was immersed in 1 mL of ice−cold 0.9% NaCl in a small beaker. The mouth of the beaker was sealed with Parafilm^©^ and then sonicated using an ultrasonic insert (Slimline−FSI−SLI 10S, Dentsply Sirona, Charlotte, NC, USA) for 90 s. This was performed on ice to compensate for the possible heat generation during the entire process. Serial dilutions (10^1^–10^6^) of the collected bacteria were prepared and plated on THB agar for *S. gordonii* and mitis–salivarius sucrose bacitracin agar (MSSB) for *S. mutans.* The plates were incubated for 48 h at 37 °C in the anaerobic chamber, after which the CFUs were counted. For the ATP assay, 100 µL of the collected bacterial solution was mixed with 100 µL of the BacTiter−Glo Microbial Cell Viability kit (Promega, Madison, WI, USA). Luminescence (Synergy HT, Biotek, Winooski, VT, USA) was measured after 5 min of incubation at room temperature.

Additionally, to complement the viability characterization of the biofilm on different surfaces, LIVE/DEAD BacLight Bacterial Viability Kit (ThermoFischer Scientific, Waltham, MA, USA) was used following the manufacturer’s instructions. After bacterial culture, samples were gently washed with 0.9% NaCl, stained with Syto−9 (green; live stain) and propidium iodide (red; dead stain), dissolved in deionized water, and incubated for 20 min in the dark. The samples were rinsed gently and then imaged on the fluorescent microscope (Eclipse E800, Nikon, Tokyo, Japan). The live cells with intact membrane stained green whereas dead cells stained fluorescent red.

**Cytocompatibility:** The cytocompatibility of the coating was tested against human dental pulp stem cells (hDPSCs). To carry this out, the cells were maintained in α−MEM media containing 10% FBS and 1% Pen−Strep at 37 °C in a humidified 5% CO_2_/95% air incubator. When the cells were 90% confluent, they were trypsinized using 0.25% trypsin/EDTA, counted, and seeded on Ti disks at a density of 5 × 10^3^ cells/sample. After 24, 72, and 144 h, samples were washed with PBS and fixed with 4% paraformaldehyde for 10 min at room temperature. Post fixation, samples were stained with 300 nM DAPI (Invitrogen, Carlsbad, CA) in PBS for 10 min. DAPI−stained cell nuclei were counted as a measure of cell numbers in each sample. After staining, the samples were washed with PBS and different field of views (FOVs) imaged on the fluorescence microscope (Eclipse E800, Nikon, Japan). Image J software (NIH, Bethesda, MD, USA) was used for counting cell nuclei from three representative images per sample and averaged over replicated samples.

**Statistics:** Data were presented graphically as mean ± standard deviation and were analyzed using GraphPad Prism. Statistical analysis was performed using 1−way ANOVA with Tukey’s multiple comparison post hoc test with *p* < 0.05 for assessing statistical significance.

## 3. Results and Discussion

### 3.1. Peptide Structures

**Figure 2:**
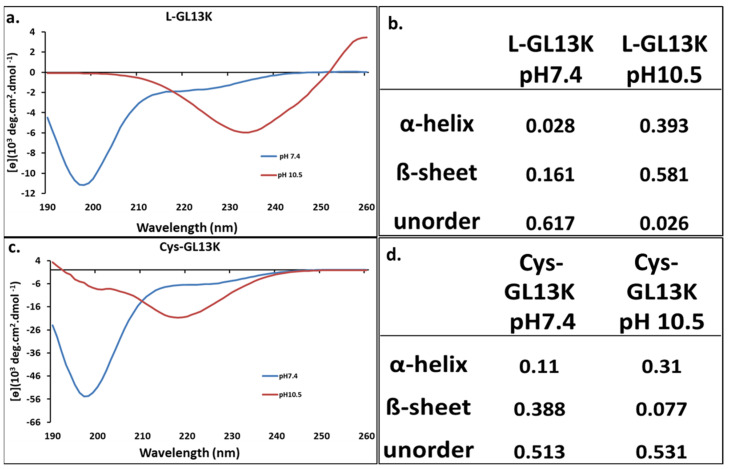
**CD analysis of L−GL13K and Cys−GL13K.** (**a**) The CD spectra and (**b**) the peptide structural components of L−GL13K. L−GL13K exhibited a molecular structure dominated by random coil at pH 7.4 and transformed to α−helix and β−sheet structures at pH 10.5. (**c**) The CD spectra and (**d**) peptide structural components of Cys−GL13K. Cys−GL13K also exhibited a molecular structure dominated by random coil at pH 7.4, but unlike L−GL13K, it mainly transformed into α−helix structures at pH 10.5.

Here, we confirmed our previous results [54,56,63] indicating that the charge screening of L−GL13K peptides by alkalinization of the peptide solution results in structural changes leading to higher molecular order, from random coil at acidic (not shown) and quasi−neutral (pH 7.4) conditions, to a notable increase in both α−helix and β−sheet structures at alkaline pH 10.5 (Figure 2). We demonstrated that the occurrence of at least one of these structural transformations was necessary for these peptides to display antimicrobial activity [63]. The introduction of the cysteine altered the Cys−GL13K peptides structure compared to L−GL13K, in that transformation into β−sheets was hindered when the pH was raised. However, transformation into α−helix structures also occurred and, thus, antimicrobial potency was expected in the Cys−GL13K peptides (34,36). We did not confirm this here, though.

### 3.2. Peptide Coating and Surface Characterization

Peptide immobilization on eTi was achieved via a thiol–maleimide click reaction. The ability to perform this chemical reaction in a short duration at a neutral pH makes it highly attractive. In comparison to our previously published reports, where GL13K peptides were covalently immobilized onto a surface via a silanization reaction using (3−chloropropyl)triethoxysilane (CPTES) as a coupling agent, the method used here offers a site−specific anchoring of the peptide when the pH is kept below 7.5. Our previously published reports highlighted that site−specific anchoring was not imperative for GL13K activity and that, under those immobilization conditions, we obtained high peptide surface coverage [64]. However, the chemoselective tethering of peptides has been associated with favored peptide orientation to increase activity and/or reduce the degradation of the peptide coating, depending on the specific peptide/biomolecule that is immobilized [65]. Therefore, in this study, we used cysteine−modified GL13K as a model antimicrobial peptide to validate the efficacy of the immobilization technique, as well as the stability and the potency of the peptide coating.

**Figure 3:**
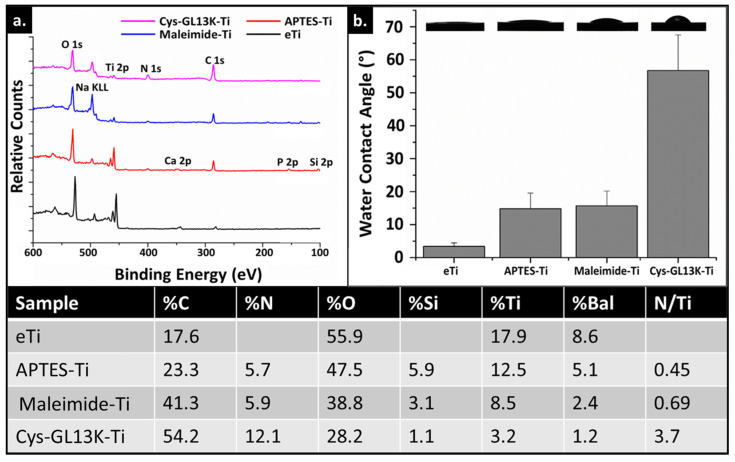
**Surface characterization of modified Ti surfaces.** (**a**) XPS survey spectra of modified Ti surfaces. Spectra of Cys−GL13K−coated Ti exhibited increase in N 1 and C 1s signals and decrease in Ti signal, compared to uncoated eTi surfaces. (**b**) Water contact angles of modified Ti surfaces with images of the dispensed sessile drops on the different substrates. Data shown are the average ± standard deviation of n = 3–6 replicates. Table shows XPS atomic % concentration (at % ratio) for the same surfaces tested in (**a**).

XPS and WCA data were used to confirm peptide immobilization on an alkaline−etch activated eTi surface. XPS surface characterization showed an increase in %N (Figure 3), concomitant with an increase in C% post silanization due to the presence of organic silanes containing an amino group on the *e*Ti surface. Then, maleimide functionalization further increased %C as expected due the organic nature of this molecule. Lastly, a notable increase of both %N and %C and so, N/Ti ratio was assessed after Cys-GL13K peptide immobilization, which demonstrated the validity of the method to obtain the antimicrobial peptide coatings. WCA supported this conclusion as the surface showed increased hydrophobicity following the successive anchoring of APTES and maleimide molecules, as they contain alkyl tails and aromatic groups, respectively. The presence of the Cys−GL13K peptides on the coating further increased the hydrophobicity of the surface due to the amphipathic nature of these peptides. However, WCA for Cys−GL13K coatings were significantly lower than for L−GL13K−modified surfaces where highly hydrophobic surfaces with WCA >110° were obtained [1,49,56,57]. This can be attributed, on the one hand, to the chemoselective nature of the reaction that might limit the ability of the amphiphilic Cys−GL13K peptides to rearrange and expose their hydrophobic residues at the solid/air interface. On the other hand, the transformation into β−sheets was inhibited for the Cys−GL13K peptides (Figure 2) and we showed that the formation of amphiphilic supramolecular self−assembled L−GL13K nanofibers in basic pH solution was associated with the formation of the β−sheet structures [54,56]. The formation of the supramolecular amphiphiles governed interactions with the comparatively hydrophilic−coated surface via hydrophilic domains in the supramolecular structures, exposing the hydrophobic regions of the structures at the solid–air and solid–liquid interface [21,56].

### 3.3. Mechanical Stability of the Peptide Coating

**Figure 4:**
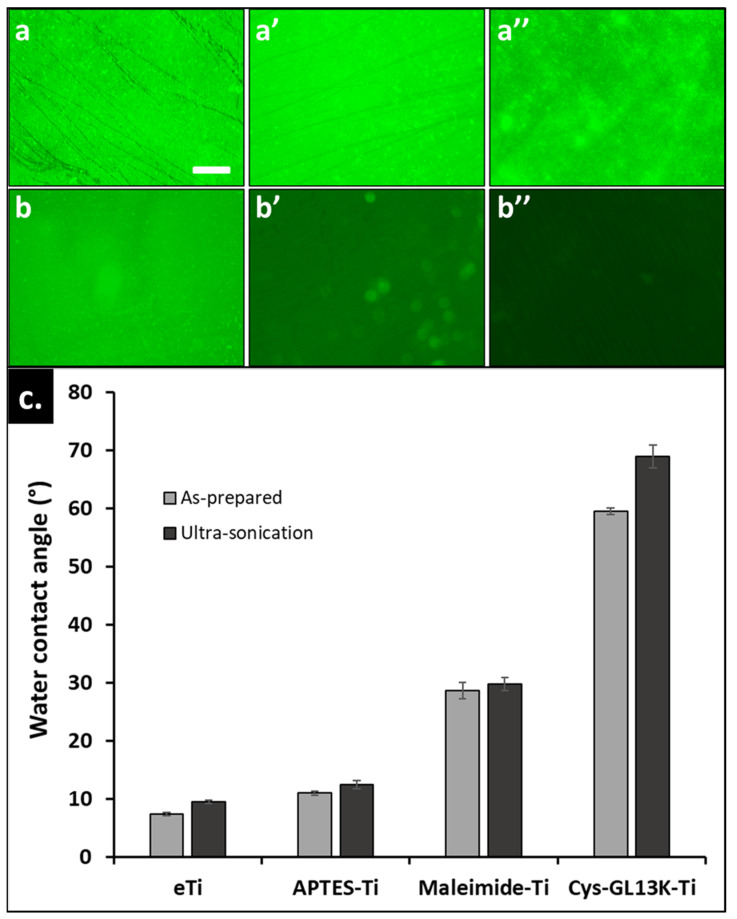
**Durability after ultrasonication of Cys−GL13K coatings.** Fluorescent micrographs of covalently anchored (top row) and physiosorbed (bottom row) Cys−GL13K onto titanium surface: (**a**,**b**) as−prepared, (**a’**,**b’**) sonication cycle of 5 min, and (**a’’**,**b’’**) sonication cycle of 30 min. The bio−conjugated peptide showed strong resistance to sonication, whereas physiosorbed coating desorbed after 30 min of sonication. (**c**) Changes in the water contact angle of covalently anchored Cys−GL13K peptides and control surfaces post sonication cycle of 30 min.

To test the mechanical stability of the covalently immobilized coatings, the coated surfaces were ultrasonicated in deionized water for short (5 min) and long (30 min) periods and the fluorescent signal of the FAM−tagged peptides was determined. On the one hand, the fluorescence signal for chemoselective cys−Gl13k−coated titanium surfaces was strong, even after 30 min of ultrasonication (Figure 4). On the other hand, a significant reduction in the signal intensity was observed for coatings obtained with physiosorbed GL13K peptides. Additionally, WCA indicated no significant change in the wettability, supporting the conclusion that the covalently anchored Cys−GL13K coatings were mechanically robust and durable. Additional work is necessary for quantifying peptide coverage on the surfaces so that we will be able to validate the durability and the efficacy of these peptide coatings with post−long−term challenges, including in more biologically relevant settings. However, our previous studies have demonstrated that the functionality of L−GL13K adsorbed onto dentin was maintained after challenging oral simulative conditions where coatings were exposed to artificial saliva for extended periods [56], or highly cariogenic biofilms [57].

### 3.4. Antimicrobial Activity of the Peptide Coating against S. gordonii and S. mutans

**Figure 5:**
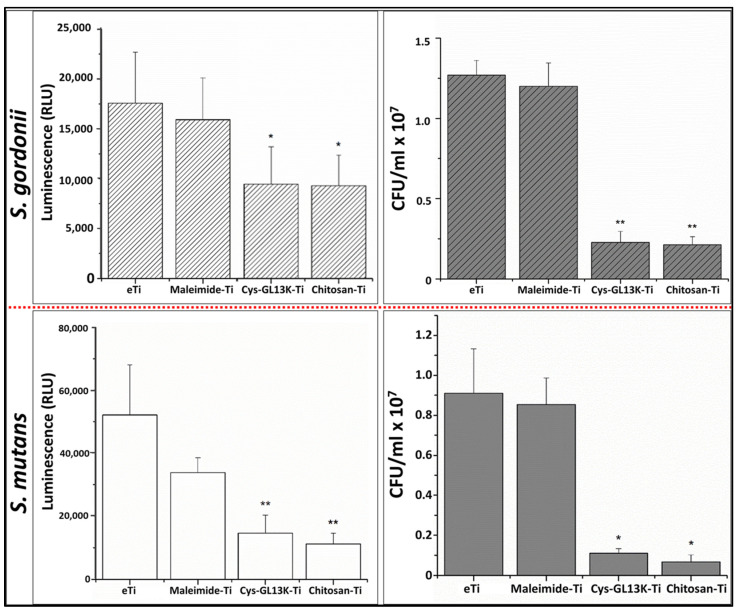
**Antibacterial activity of cys−GL13K−coated and control titanium surfaces against *S. gordonii* (top) and S. mutans (bottom) biofilms.** Vitality of bacteria was assessed by ATP bioluminescence (left graphs), as a measure of metabolic activity, and bacteria viability was assessed by counting colony forming units (CFUs; right graphs). Significant differences between groups are marked * (*p*−value < 0.05) and ** (*p*−value < 0.01).

**Figure 6:**
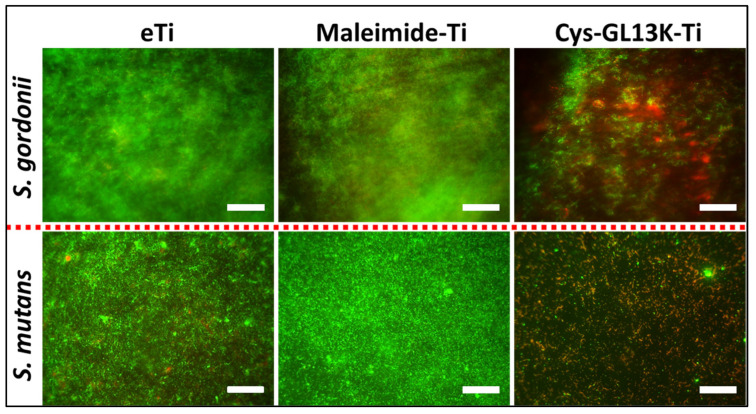
**LIVE/DEAD imaging of *S. gordonii* (top images) and S. mutans (bottom images) biofilms on cys−GL13K−coated and control titanium surfaces**. Scale is 100 µm.

*Streptococcus* species play important roles in biofilm formation and the pathogenesis of tissues of the oral cavity [66]. *S. mutans* has been described as a common pathogenic species present in biofilms leading to dental decay. *S. gordonii* is a primary colonizer on oral surfaces that provides attachment for the subsequent pathogenic biofilm formation by *Porphyromonas gingivalis* [67], implicated in periodontal and peri−implant infections [67]. Cys−GL13K coatings displayed a significant reduction in the viability (CFUs) and vitality (ATP bioluminescence) of both *S. mutans* and *S. gordonii* biofilms compared to non−coated eTi and maleimide−coated Ti control surfaces (Figure 5). The LIVE/DEAD fluorescent imaging test (Figure 6) confirmed that cys−GL13K coatings on titanium had strong antimicrobial effects with dominant fluorescent red bacteria, indicating that these bacteria had compromised membranes. However, biofilms on eTi and maleimide−coated titanium had dominant fluorescent green viable bacteria with intact membranes. Taken together, these results supported the conclusion that the chemoselective immobilization of L−GL13K peptides on Ti surfaces using N−terminus single−cystine addition resulted in potent antimicrobial coatings against relevant bacteria strains in the oral cavity. The release of Cys−GL13K peptide from the surfaces cannot be fully discarded and further work will include an investigation of the efficacy of these coatings in environments that more closely simulate oral challenges, such as those posed by multispecies or microcosm biofilms and/or under dynamic media flows in CDC or drip flow bioreactors.

### 3.5. Cytocompatibility of the Peptide Coating with hDPSCs

**Figure 7:**
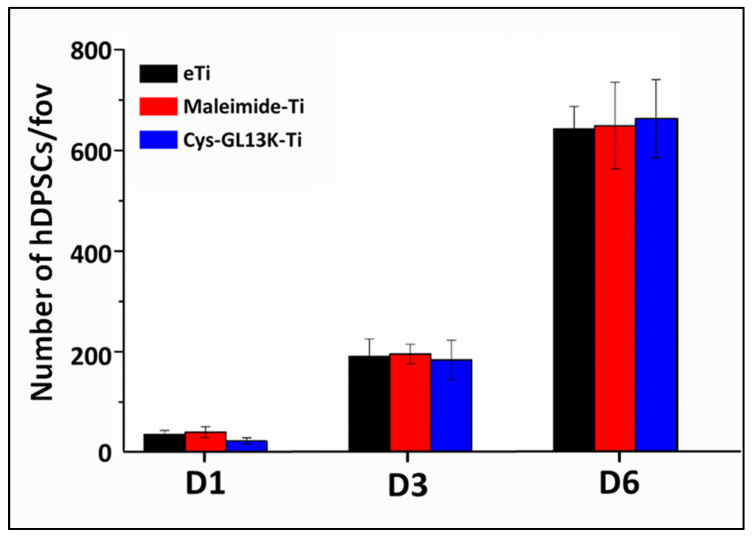
**Cytocompatibility with hPDSCs of cys−GL13K−coated and control titanium surfaces.** D1, D3, and D6: day 1, day 3, and day 6 of hDPSCs culture, respectively. fov: field of view.

We finally assessed cytocompatibility of the cys−GL13K peptide−coated surfaces compared to that of the eTi− and maleimide−coated titanium control surfaces against hDPSCs. Our long−term goal is to prepare multifunctional coatings on dental implants with a specific spatial orientation to prevent infection, without compromising bone healing. Therefore, we assessed cell attachment and the proliferative capability of hDPSCs by counting the number of nuclei on different surfaces over a period of 6 days. The presence of the antimicrobial peptide coating did not interfere with the proliferation of hDPSCs as cell numbers continuously increased over culture time, with no significant differences observed between different surfaces (Figure 7). This observation agrees with our previously published reports where L−GL13K−coated surfaces supported cell proliferation and/or function relative to the uncoated titanium controls [1,21,34,49]. Future work will focus on assessing the cell function of the coated surfaces towards the osteogenic differentiation of hDPSCs in vitro and bioactivity in vivo.

## 4. Conclusions

We successfully functionalized titanium surfaces with cysteine−terminated GL13K using a maleimide–thiol−mediated Michael−type addition reaction. We further confirmed the stability of the peptide coating when mechanically challenged. Our in vitro studies confirmed the antibacterial potency of the chemoselective cys−GL13K peptide−coated surface against relevant oral bacteria with high cytocompatibility with hDPSCs. Additional work will focus on translating this immobilization chemistry to other functional peptides like angiopoietin−1 for enhanced bone regeneration around dental implants, or LamLG3 peptides for soft−tissue attachment around abutments and dental implants.

## Data Availability

The data can be made available upon a direct request to the authors.
